# Liver Imaging and Data System (LI-RADS) Version 2018 and Other Imaging Features in Intrahepatic Cholangiocarcinoma in Chinese Adults with vs. without Chronic Hepatitis B Viral Infection

**DOI:** 10.1155/2021/6639600

**Published:** 2021-03-04

**Authors:** Ying-ying Liang, Shuo Shao, Sichi Kuang, Jingbiao Chen, Jing Zhou, Bingjun He, Linqi Zhang, Yao Zhang, Kathryn J. Fowler, Jin Wang

**Affiliations:** ^1^Department of Radiology, The Third Affiliated Hospital of Sun Yat-Sen University, 600 Tianhe Rd., Guangzhou 510630, Guangdong, China; ^2^Department of Radiology, Guangzhou First People's Hospital, School of Medicine, South China University of Technology, Guangzhou, China; ^3^Shandong Medical Imaging Research Institute, Shandong University, Jinan, Shandong, China; ^4^Department of Radiology, Jining No. 1 People's Hospital, Jining, Shandong, China; ^5^Liver Imaging Group, Department of Radiology, University of California, San Diego, La Jolla, CA, USA

## Abstract

**Purpose:**

To describe liver imaging reporting and data system (LI-RADS) version 2018 and other MRI imaging features in intrahepatic mass-forming cholangiocarcinoma (iCCA) in Chinese adults with vs. without chronic hepatitis B viral (HBV) infection.

**Methods:**

We retrospectively enrolled 89 patients with pathologically proven iCCA after multiphase imaging performed between 2004 and 2017 at a tertiary medical center in southern China. Based on whether patients had chronic HBV, iCCA was divided into two subgroups: HBV-positive (*n* = 50 patients, including 9 with cirrhosis) vs. HBV-negative (*n* = 39 patients, including 14 with hepatolithiasis and 25 with no identifiable risk factor for iCCA; none had cirrhosis). Two independent abdominal radiologists in consensus reviewed the largest mass in each patient to assign LI-RADS v2018 features; they also scored each observation's shape and location. Imaging features were compared using chi-square or Fisher's exact tests.

**Results:**

Most iCCAs in HBV-positive (88% (44/50)) and HBV-negative (97% (38/39)) patients had at least one LR-M feature. Compared to iCCAs in HBV-negative patients, iCCAs in HBV-positive patients were more likely to have at least one major feature of HCC (46% (23/50) vs. 8% (3/39), *P* < 0.001) and more likely to be smooth (42% (21/50) vs. 10% (4/39), *P* = 0.001). Six of 50 (12%) iCCAs in HBV-positive patients and 1/39 (3%) iCCAs in HBV-negative patients had at least one major feature of HCC without any LR-M feature.

**Conclusions:**

In this retrospective single-center study in Chinese adults, iCCAs in HBV-positive patients were more likely to resemble HCCs than iCCAs in HBV-negative patients.

## 1. Introduction

Intrahepatic mass-forming cholangiocarcinoma (iCCA), a malignancy characterized by cells resembling bile duct epithelium, is the second most common intrahepatic malignancy behind hepatocellular carcinoma (HCC) and accounts for approximately 15% to 20% of all primary liver cancers [1–4]. Although still relatively uncommon, iCCA has increased in incidence by up to 165% in the last 30 years in the U.S.A. [5], and even higher rates have been reported in Thailand, China, and other parts of Asia [6]. Surgical resection remains the only curative treatment; however, the prognosis even after curative resection is unsatisfactory with a five-year survival rate of 11%–44% [7–11].

Risk factors for iCCA depend on geographic region. In China and other East Asian countries, common risk factors include chronic hepatitis B viral (HBV) infection, hepatolithiasis, and liver fluke infestation, although many iCCAs arise in patients without any identifiable risk factors [12–16]. Chronic HBV infection also predisposes to HCC development [17], meaning that both iCCA and HCC may arise in the same patient population. Differentiation between iCCA and HCC in such patients is important since the tumors differ in prognosis and management [18, 19].

Prior studies have described MR imaging features characteristic of iCCA [20–24], collectively termed “targetoid” by the Liver Imaging Reporting And Data System (LI-RADS) [25]. The presence of even one such feature is thought to indicate a substantial possibility of malignancy other than HCC. The association between targetoid features and iCCA, however, has been studied mainly in patients without HBV or other risk factors for HCC [25]. The imaging appearance of iCCAs in HBV-positive patients is not well understood, casting doubt on the applicability of targetoid features in such patients. In addition to targetoid imaging features, LI-RADS also lists infiltrative appearance, marked diffusion restriction, and necrosis/ischemia as features of LR-M [25]. The purpose of this study was to describe and compare LI-RADS version 2018 and other MRI imaging features of iCCAs in HBV-positive vs. HBV-negative Chinese adults. A secondary purpose was to determine how these features may impact LI-RADS categorization in the HBV-positive cohort.

## 2. Materials and Methods

### 2.1. Patients

Our institutional review board approved and waived the requirement for written informed consent for a retrospective review of a prospectively maintained pathology database of all patients with iCCA treated at our institution, a tertiary care center in the southern China, between April 2004 and March 2017. After reviewing the electronic medical records of all patients in the database, we enrolled in our retrospective study all patients who met the following eligibility criteria: inclusion—(1) pathologically proven diagnosis of iCCA and (2) abdominal MR scans performed within 2 months prior to a histologic diagnosis and with satisfactory image quality as judged retrospectively by an abdominal radiologist (Y. L. with 7 years of experience in abdominal MRI). Exclusion: (1) combined hepatocellular cholangiocarcinoma (*n*=24); (2) hilar and exhepatic cholangiocarcinoma (*n* = 119), or (3) no preoperative MRI examination (*n*=49).

### 2.2. Pathologic Analysis

The histopathologic diagnosis of iCCA was confirmed by surgical resection (*n*=59) or percutaneous liver biopsy (*n*=30). For all specimens, the diagnosis was based on a combination of histopathology and immunohistochemical staining as deemed appropriate and if sufficient tissue was available in the setting of biopsy. All original hepatic specimens were reviewed by a hepatic pathologist with more than 14 years of experience in hepatic pathology who was blinded to the imaging findings. Any disagreement was resolved by discussion to arrive at a consensus.

### 2.3. Clinical Data Collection

The electronic medical records of all patients were retrospectively reviewed. The values of serum biochemical tests and the following serum tumor markers were extracted if they were collected for clinical care before treatment and within 1 week before or after the selected MRI exam: alpha-fetoprotein (AFP), carcinoembryonic antigen (CEA), carbohydrate antigen 19-9 (CA19-9) and carbohydrate antigen 125 (CA125).

### 2.4. MR Image Acquisition

Patients were scanned supine on a 3 T (Discovery MR750, GE Healthcare, Milwaukee, WI) or 1.5 T (GE Signa EXCITE HDxt, GE Healthcare, Waukesha, WI; Philips Achieva, Best, The Netherlands) whole-body MR scanner with an eight-channel phased-array coil centered over the abdomen. Precontrast pulse sequences included breath-hold coronal fast imaging employing steady-state acquisition (FIESTA), breath-hold coronal single-shot fast spin-echo (SSFSE), respiratory-triggered axial T2-weighted fast spin-echo (FSE), breath-hold two-dimensional dual-echo T1-weighted gradient-recalled-echo images at nominal out-of-phase (1.15 ms at 3 T, 2.3 ms at 1.5 T) and in-phase (2.3 ms at 3 T, 4.6 ms at 1.5 T) echo times, and respiratory triggered axial diffusion-weighted spin-echo echo-planar imaging with two *b* values (*b* = 0 and 800 sec/mm^2^). Breath-hold 3D T1W gradient-recalled-echo imaging (liver acquisition with volume acceleration (LAVA) on GE and T1 high resolution isotropic volume excitation (THRIVE) on Philips) were performed before and at multiple time points dynamically after injection of Gd-BOPTA (Gadobenate dimeglumine, Bracco), Gd-DTPA (Gadopentetate dimeglumine, Bayer), and Gd-EOB-DTPA (Gadolinium ethoxybenzyldiethy-lenetriaminepentaacetic acid, Bayer). A dual-arterial phase (AP) was initiated 15–20 secs after contrast media arrival in the distal thoracic aorta using bolus triggering, a dual portal venous phase (PVP) was acquired at 1 minute after contrast injection, and a delayed phase (DP)/transitional phase (TP) was acquired at 3 minutes. Hepatobiliary phase (HBP) images were also acquired in patients receiving Gd-EOB-DTPA and in some patients receiving Gd-BOPTA; as explained as follows, such images were not analyzed.

### 2.5. Image Analysis

If there was more than one MR exam within the acceptable time window, the one most immediately proximate to histology sampling was selected. MR images were evaluated independently by two abdominal radiologists Y. L. and S. S. with 7 and 8 years of experience in abdominal radiology, respectively. The reviewers knew that the patients had iCCA but were blinded to the patients' history and laboratory results. Discordance on all features and LI-RADS category between the two was resolved by consensus which will be used in the final statistical analysis. Before reviewing the MR images, each reader was provided a 1-hour lecture and an additional 1-hour hands-on instruction with 10 training cases on LI-RADS v2018 features and categories by the third senior abdominal radiologist J. W. The training cases were selected from patients not included in the study population.

The reviewers were instructed to identify and characterize the largest lesion in each liver with respect to all LI-RADS v2018 imaging features (https://www.acr.org/Clinical-Resources/Reporting-and-Data-Systems/LI-RADS/CT-MRI-LI-RADS-v2018) as well as the following additional features defined in Table 1: lobe (left/right/both), location (subcapsular, not subcapsular), shape (round, lobulated/irregular), and presence of absence of liver surface retraction, hepatic lobar atrophy, satellite nodules, and lymph node metastases. We did not assess bile duct obstruction, a feature of iCCA [22] because a substantial proportion of the HBV-negative patients had underlying hepatolithiasis, a condition in which bile duct obstruction precedes iCCA formation rather than being caused by it.

LI-RADS v2018 imaging features included (a) features of LR-M (rim arterial phase hyperenhancement (APHE), peripheral “washout,” delayed central enhancement, targetoid appearance on DWI, infiltrative appearance, marked diffusion restriction, and necrosis/severe ischemia); (b) major features of HCC (nonrim APHE, nonperipheral “washout,” and enhancing “capsule”); (c) ancillary features favoring malignancy in general (corona enhancement, fat sparing in solid mass, restricted diffusion, mild-moderate T2 hyperintensity, and iron sparing in solid mass); (d) ancillary features favoring HCC in particular (intralesional fat, blood products, nodule-in-nodule and mosaic architecture); and size (tumor diameter). The mean size measured by the two abdominal radiologists was calculated for each mass. “Threshold growth” was not scored as prior exams were not reviewed. Ancillary features favoring benignity were not scored because the two abdominal radiologists were aware that this study included only malignant tumors. Due to the inconstant use of hepatobiliary agents, imaging features evaluable only with these (transitional phase hypointensity, HBP hypointensity, and HBP isointensity) were not analyzed.

Based on the consensus imaging feature scores and mean size, each iCCA in the HBV-positive group was assigned a LI-RADS v2018 diagnostic category. LI-RADS categories were not assigned to iCCAs in the HBV-negative patients, for whom LI-RADS does not apply.

### 2.6. Statistical Analysis

For analysis purposes, patients were divided into two groups: iCCA in patients with chronic HBV infection and iCCA in patients without chronic HBV. Patient characteristics were summarized. Interobserver agreement for individual features was assessed; kappa (*κ*) values was reported. The results were interpreted as slight agreement for *κ* values of 0.01–0.20, fair agreement for 0.21–0.40, moderate agreement for 0.41–0.60, substantial agreement for 0.61–0.80, and excellent agreement for 0.81–1.00. Continuous variables were expressed as means ± standard deviations and compared with Student's *t*-test or Mann–Whitney *U*-test as appropriate. Categorical variables were expressed as number and percentages and compared by chi-square or Fisher's exact tests. Statistical significance was defined as a *P* value of less than 0.05. All analyses were performed with software SPSS (version 20.0 for Windows; SPSS, Chicago, IL, USA).

## 3. Results

### 3.1. Patient Characteristics

**Figure 1:**
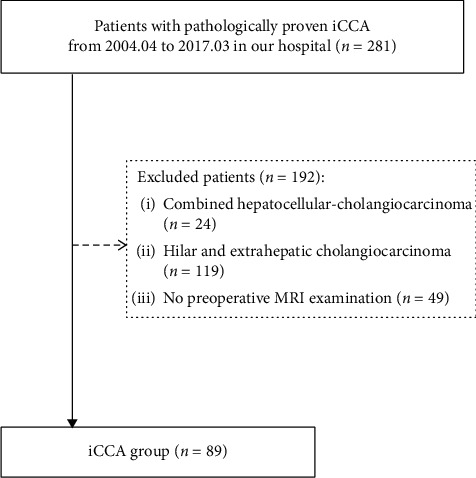
Flow diagram of the study population.

Eighty-nine patients with a diagnosis of iCCA (M : *F* = 57 : 32) met eligibility criteria and were enrolled (Figure 1). Patients were divided into two groups: iCCA in patients with chronic HBV infection (HBV-positive, *n* = 50) and iCCA in patients without chronic HBV (HBV-negative, *n* = 39). The former group included 9 patients with cirrhosis. The latter group included 14 patients with hepatolithiasis and 25 patients with no attributable cause (cryptogenic); no HBV-negative patient had cirrhosis. Patient characteristics are summarized in Table 2. Compared with HBV-negative patients, HBV-positive patients were younger (*P* = 0.007) and more likely to be male (*P* < 0.001); they also had lower serum CA19-9 (*P* = 0.034) and serum globulin (*P* = 0.025) levels, but higher AFP levels (*P* = 0.001). iCCA lesions were smaller in HBV-positive than HBV-negative patients (mean size: 52.6 ± 32.8 mm vs. 69.5 ± 26.2 mm, *P* = 0.006). Other differences were not significant.

### 3.2. MR Imaging Features

Table 3 summarizes consensus MR imaging features in HBV-positive vs. HBV-negative patients.

#### 3.2.1. LR-M Features

Interreader agreement for all LR-M features was moderate (*κ* = 0.41–0.55), except for peripheral “washout,” which achieved fair interreader agreement (*κ* = 0.36).

**Figure 2:**
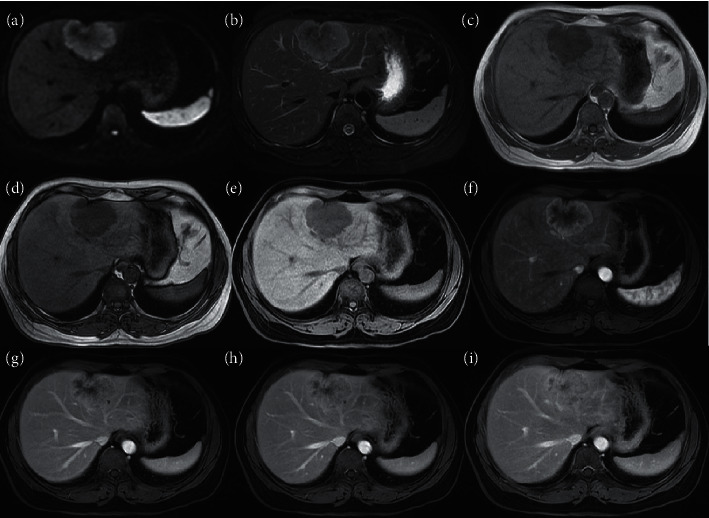
MR images in a 48-year-old HBV-negative woman with iCCA confirmed by surgical resection. (a) DWI image; (b) T2WI; (c) T1WI image in-phase; (d) T1WI image out-phase; and 3D fat-suppressed T1WI precontrast (e) and in the (f) arterial, (g) portal phase, and (h, i) delayed phases. The tumor measures 49 × 65 mm, retracts the liver surface, and shows targetoid appearance on DWI, rim APHE, delayed central enhancement, and peripheral washout appearance.

**Figure 3:**
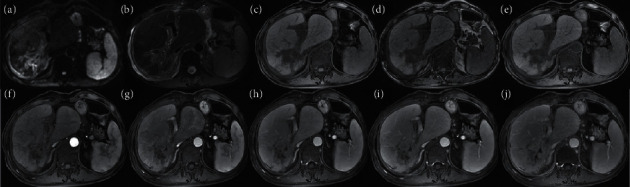
MR images in a 68-year-old HBV-negative man with iCCA confirmed by surgical resection. (a) Diffusion-weighted image; (b) T2-weighted image; (c) T1-weighted in-phase image; (d) T1-weighted out-of-phase image; and (e) T1-weighted images precontrast and in the (f) early arterial, (g) late arterial, (h) early portal venous, (i) late portal venous phases, and (j) delayed phases. The tumor measures about 60 × 88 mm and shows infiltrative appearance with delayed central enhancement.

Based on consensus scores, most iCCAs in HBV-positive (88% (44/50)) and HBV-negative (97% (38/39)) patients had at least one LR-M feature; these differences were not significant (*P* = 0.103). The most common individual features in both groups were delayed central enhancement (82–95%), targetoid appearance on DWI (62–72%), rim APHE (62–69%), and necrosis or severe ischemia (60–67%) (Figure 2). Infiltrative appearance was more frequent in iCCAs in HBV-negative than HBV-positive patients (54% (21/39) vs. 22% (11/50), *P* = 0.002) (Figure 3). Other individual LR-M features did not differ significantly in frequency between groups (*P* ≥ 0.10 for all).

#### 3.2.2. Major Features

**Figure 4:**
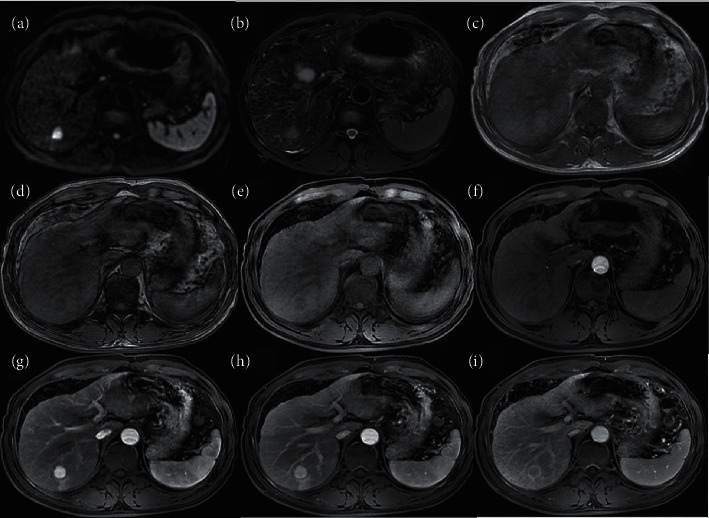
MR images in a 46-year-old HBV-positive man with iCCA confirmed by surgical resection, and this case was miscategorised as LR-5. (a) DWI image; (b) T2WI; (c) T1WI image in-phase; (d) T1WI image out-phase; and (e) 3D fat-suppressed T1WI precontrast and in the (f) early arterial, (g) later arterial phase, (h) portal phase, and (i) delayed phases. The tumor measures 17 × 17 mm and shows APHE, nonperipheral “washout” appearance and capsule.

Interreader agreement for all major features was fair (*κ* = 0.31–0.35). Based on consensus scores, iCCAs in HBV-positive patients were more likely to have at least one major feature of HCC than iCCAs in HBV-negative patients (46% (23/50) vs. 8% (3/39), *P* < 0.001). Enhancing “capsule” (16%, 14/89) and nonrim APHE (13%, 12/89) were the two most frequent major features, seen in of iCCAs overall. Nonrim APHE was more frequent in iCCAs in HBV-positive than in HBV-negative patients (24% (12/50) vs. 0% (0/39), *P* = 0.001) (Figure 4). Other major features did not differ significantly in frequency between groups (*P* ≥ 0.05 for all).

#### 3.2.3. Ancillary Features

Interreader agreement for all ancillary features was excellent (*κ* = 0.86–0.95). Based on consensus scores, two ancillary features favoring malignancy in general (restricted diffusion and mild-moderate T2 hyperintensity) were present in all (89/89) iCCAs, regardless of patients' HBV status. Thus, all iCCAs in each group had at least two ancillary features favoring malignancy in general. Surprisingly, four iCCAs (4/50, 8%) in HBV-positive patients and one iCCA (1/39, 3%) in HBV-negative patients had at least one ancillary feature favoring HCC in particular (either intralesional fat or blood products). No iCCA had nodule-in-nodule architecture, mosaic architecture, or nonenhancing “capsule.”

With regard to individual ancillary features, one feature favoring malignancy in general (corona enhancement) was more frequent in iCCAs in HBV-negative than HBV-positive patients (77% (30/39) vs. 36% (18/50), *P* < 0.001). Other ancillary features did not differ significantly in frequency between groups (*P* ≥ 0.10 for all).

#### 3.2.4. Other Features

Interreader agreement was excellent for liver surface retraction (*κ* = 0.83), moderate for lesion shape (*κ* = 0.79) and location (*κ* = 0.76), and fair for hepatic lobe atrophy, satellite nodules, and lymph node metastasis (*κ* = 0.31–0.37). Compared to iCCAs in HBV-negative patients and based on consensus scores, iCCAs in HBV-positive patients were more likely to be smooth (42% (21/50) vs. 10% (4/39), *P* = 0.001) but less likely to be subcapsular (68% (34/50) vs. 95% (37/39), *P* = 0.002) or associated with liver surface retraction (22% (11/50) vs. 44% (17/39), *P* = 0.03).

### 3.3. LI-RADS Categorization of iCCAs in HBV-Positive Patients

Of the 50 iCCAs in HBV-positive patients, 44 (88%) were correctly categorized as LR-M (*n* = 40) or LR-TIV associated with a LR-M parenchymal mass (*n* = 4); six (12%) were miscategorized as LR-5 (*n* = 4), LR-4 (*n* = 1), or LR-3 (*n* = 1) (Table 4). Of those miscategorized, 3 of 6 iCCAs were diagnosed by biopsy. Compared to the 44 correctly categorized iCCAs, the six miscategorized iCCAs were smaller (20.4 ± 8.1 vs. 57.8 ± 29.9 mm, *P* = 0.002), more frequently <20 mm (50% (3/6) vs. 5% (2/44), *P* = 0.006), and more frequently smooth (100% (6/6) vs. 34% (154/44), *P* < 0.001). None of these observations demonstrated any LR-M features. There was no difference in location. As mentioned earlier, LI-RADS categories were not formally assigned for iCCAs in HBV-negative patients. Nevertheless, no iCCA in an HBV-negative patient had nonrim APHE, and therefore, none would have met LR-5 criteria. Moreover, of the three HBV-negative iCCAs with at least one major feature of HCC, two of three also had at least one LR-M feature. Thus, there was only 3% (1/39) iCCA in an HBV-negative patient with at least one major feature of HCC but without any LR-M feature.

## 4. Discussion

Our study shows that iCCAs display similar imaging features in Chinese adults, regardless of whether the tumors arise in patients with or without underlying HBV infection: targetoid features manifest with high frequency in both populations. There was a trend toward less frequent LR-M features and more frequent HCC major features in the HBV-positive cohort, however, and a nonnegligible proportion (8%) of iCCAs arising in HBV-positive patients were miscategorized as LR-5 and another 4% were miscategorized as LR-4 or LR-3.

LI-RADS category 5 observations are assigned on the basis of major features. We evaluated the frequency of three of the four LI-RADS major features of HCC: nonrim APHE, nonperipheral “washout,” and enhancing “capsule.” Nonrim APHE is perhaps the most essential feature of HCC, as without its presence, an observation cannot be categorized as LR-5. We found that 24% (12/50) of iCCAs arising in the HBV-positive population had nonrim APHE. LI-RADS v2018 prescribes specific features that when present are sufficient for diagnosis as LR-M. If both LR-5 and LR-M features are seen in the same lesion, the appropriate category is LR-M. Recognition that nonrim APHE is seen in about one quarter of iCCAs arising in HBV-positive patients emphasizes the importance of evaluating and strictly applying all LR-M features, not just rim APHE.

Of note, capsule appearance was one of the more common major features seen in our iCCA population. While capsule has historically been considered a highly specific feature of HCC, our findings suggest that it may not be as rare as once thought in iCCAs, particular in HBV-positive patients. Capsule appearance (16%, 14/89) in our study was also reported by Horvat et. (16%–49% for four readers, 8/51–25/51) [26] and Ni et al. (34.8%, 48/138) [27], but it was more common in HCC. This could be in part explained by the emerging hypothesis that iCCAs are not entirely distinct or “pure” entities, but rather exist along a genophenotypic spectrum exhibiting at times features that overlap with hepatocellular carcinomas [28]. Further multicenter studies are warranted in HBV-positive patients.

LR-M features are divided into targetoid and nontargetoid features. Targetoid appearance is one of the most important features in the discrimination of LR-M from other categories according to LI-RADS v2018. This imaging appearance is thought to reflect the underlying pathology of iCCAs and other non-HCC malignancies, which tend to have greater cellularity and vascularity in the periphery and looser edematous fibrotic stroma centrally [29, 30]. Consistent with previous studies [3, 24, 31], the most prevalent enhancement patterns of 89 iCCAs in our study included two manifestations of targetoid dynamic enhancement appearance: rim APHE (58/89, 65%), and delayed central enhancement (78/89, 88%), without significant differences between the two risk groups. Interestingly, peripheral “washout” was uncommon in our cohort and achieved only fair interreader agreement. Targetoid appearance on DWI was relatively common, being present in 59 of 89 (67%) iCCAs overall. This proportion is similar to that reported by Joo et al. (24/35, 68.6%) [32] but slightly lower than that reported by Park et al. (24/32, 75.0%) [33] and Min et al. (66/79, 83.5%) [34]. The fifth manifestation of targetoid appearance (targetoid appearance in the transitional or hepatobiliary phase) was not assessed in this study due to the inconstant use of hepatobiliary agents.

For malignant lesions without targetoid appearance, LI-RADS suggests the use of other features including infiltrative appearance, marked diffusion restruction, and necrosis/severe ischemia. In our study, the frequency of infiltrative appearance differed significantly across the two groups (*P* < 0.001), being most common in iCCAs in HBV-negative patients. Necrosis or severe ischemia was common (60–67%) in our cohort and equally seen in HBV-positive and HBV-negative cohorts. By comparison, marked diffusion restriction was very common in both groups (100%).

With regards to ancillary features, a few interesting results merit discussion. Corona enhancement was significantly more common in iCCAs in HBV-negative than HBV-positive patients. It has been reported that corona enhancement might favor the diagnosis of HCC and convey information on microvascular invasion and metastatic satellites [35–37]. Our study shows that corona enhancement can occur in non-HCC malignancies. Further research is needed to determine whether the presence of corona enhancement in iCCA has similar prognostic implications as in HCC. With regards to ancillary features favoring HCC, we found that intralesional fat and blood products were detected occasionally (1/89, 1.1% for fat, 4/89, 4.5% for blood), which is comparable with the results of other studies [20, 38]. Since iCCAs do not possess cellular mechanisms associated with lipid uptake, further research is needed to confirm that intralesional fat can accumulate in iCCAs.

In addition to LI-RADS features, we evaluated seven other features (lobe, location, shape, liver surface retraction, hepatic lobe atrophy, satellite lesions, and lymph node metastasis) that may be applied as suggestive features of non-HCC malignancy by radiologists in practice. Of note, a substantial proportion (42%) of iCCAs in HBV-positive patients had a smooth shape, potentially resembling HCC morphologically.

LI-RADS diagnostic accuracy is a clinically relevant topic because many liver lesions, even hepatic tuberculosis [39, 40], can display a contrast-enhanced LI-RADS features resembling HCC. The iCCAs diagnosis might be more challenging in HBV-positive patients due to the overlapping imaging patterns compared with HCC. This is linked to rich tumor cells and no central necrosis which lead to APHE and washe out in small iCCAs, especially lesions ≤20 mm [41–43]. Miscategorization iCCAs as LR-5 can be problematic in geographic regions where liver transplant without biopsy confirmation may be offered as front-line therapy for early-stage HCC. In regions where resection is the primary treatment modality, the distinction for small lesions may be less important as the treatment would remain the same. Further research is needed to identify features and other factors to further reduce the risk of miscategorization of iCCAs as LR-5.

Our study has limitations. First, retrospective design produces selection bias. Therefore, more prospective research is needed to confirm the results in the future. Second, our reference standard is imperfect with some patients being diagnosed with biopsy rather than complete surgical resection, and of the 50 iCCAs in HBV-positive patients, 6 (12%) were miscategorized as LI-RADS 3, 4, or 5, and 3 of those were confirmed by percutaneous liver biopsy. It is possible that these tumors may have represented combined hepatocellular-cholangiocarcinomas, but due to sampling error, only the iCCA portion was sampled. Third, although we investigated the concept that iCCA may appear differently in patients at high risk for HCC vs. those without risk factors, our high-risk population consisted solely of HBV-positive patients. Hence, our results are likely not generalizable to Western cohorts where the high-risk population would be expected to have a higher frequency and severity of cirrhosis, in which altered vascular supply and background parenchymal heterogeneity could affect imaging appearances. The differences in appearance of iCCAs between cirrhotic livers and noncirrhotic livers should be the focus of future research studies. Similarly, compared to Western population, our HBV-negative cohort included a relatively high proportion of patients with hepatolithiasis, which by definition are associated with bile duct obstruction, precluding the analysis of this imaging feature.

In conclusion, LR-M features are present in similar frequencies in iCCA in patients with HBV as those without HBV. However, small iCCA arising in the setting of HBV may be more likely to show major features of HCC, such as nonrim APHE, leading to a low but not negligible risk of miscategorization as LR-5.

## Figures and Tables

**Table 1:** Other MR imaging features and their definitions.

Parameters	Definition
Lobe	*Left* (tumor entirely contained within left lobe)
	*Right* (tumor entirely contained within right lobe)
	*Both* (tumor extending into both lobes)
Location	*Subcapsular* (no visible parenchyma between mass and liver surface)
	*Not subcapsular* (visible parenchyma between mass and liver surface)
Shape	*Round* (circular or oval without lobulation or irregularity)
	*Lobulated/irregular* (lobulated and/or irregular margin)
Liver surface retraction	Liver border retraction peripheral to tumor
Hepatic lobe atrophy	Unequivocal decrease in lobar volume with crowding of the bile ducts and hepatic vasculature
Satellite nodules	Presence of discrete parenchymal nodules within 1 cm of primary tumor margin
Lymph node metastases	Considered positive when the short axis of a porta hepatis lymph node was greater than 10 mm or when a node showed central necrosis on MRI

**Table 2:** Patient clinical characteristics in the HBV-iCCA and iCCA with no high-risk.

	HBV-positive group (*n*=50)	HBV-negative group (*n*=39)	*P* value
*Mean age (years)*	50.2 ± 11.2 (23–70)	57.0 ± 10.7 (25–74)	0.007
*Sex ratio (M:F)*	41 : 9	16 : 23	<0.001
*MRI-histology interval (days)*	6.8 ± 7.9 (1–14)	7.0 ± 7.4 (1–15)	0.909
*Cirrhosis*	9 (18%)	0	0.005
*Size (mm), mean ± SD, (range)*	52.6 ± 32.8 (9–134)	69.5 ± 26.2 (25–127)	0.006
*Tumour markers*			
AFP (ng/mL)	96.2 ± 273.2 (0.2–1210)	3.2 ± 1.7 (0.4–8.4)	0.001
CA19-9 (*μ*/mL)	2.8 ± 12.4 × 10^3^ (2–83.4 × 10^3^)	51.9 ± 205.9 × 10^3^ (2–1200 × 10^3^)	0.034
CEA (*μ*g/mL)	2.9 ± 4.2 (0.5–25.6)	12.6 ± 28.1 (0.5–100)	0.198
CA12-5 (*μ*/mL)	59.3 ± 99.7 (4.8–464.1)	308.0 ± 997.3 (5.1–2193.1)	0.182
*Biochemical markers*			
AST (U/L)	42.1 ± 40.0 (12–200)	51.8 ± 63.9 (16–273)	0.788
ALT (U/L)	33.8 ± 24.0 (12–115)	52.8 ± 77.5 (6–354)	0.875
ALB (g/L)	40.2 ± 6.1 (18.4–50.8)	40.1 ± 5.0 (27.8–47.9)	0.869
GLB (g/L)	28.2 ± 5.4 (20.3–39.4)	30.5 ± 3.9 (21.8–37.7)	0.025
T-BIL (*μ*mol/L)	21.9 ± 40.9 (5.3–300.4)	45.5 ± 76. 5 (5.9–303.9)	0.836
D-BIL (*μ*mol/L)	10.4 ± 31.0 (1.04–222.9)	30.2 ± 61.3 (1.3–237.6)	0.363

HBV chronic hepatitis B virus infection; iCCA, mass-forming intrahepatic cholangiocarcinoma; AFP, alpha-fetoprotein; CA19-9, carbohydrate antigen 19–9; CEA, carcinoembryonic antigen; CA12-5, carbohydrate antigen 12–5; ALT, alanine aminotransferase; AST, aspartate aminotransferase; GGT, gamma-glutamyl transpeptidase; TPROT, total protein; ALB, Albumin; GLB, globulin; T-BIL, total bilirubin; D-BIL, direct bilirubin. Continuous variables expressed as means ± standard deviations with ranges in parentheses or as counts with percentages in partentheses, as appropriate.

**Table 3:** Consensus MR imaging features of of iCCA in HBV-positive and HBV-negative patients.

	Imaging features	HBV-positive group (*n*=50)	HBV-negative group (*n*=39)	*P* value
LR-M features	Rim APHE	31 (62%)	27 (69%)	0.477
	Peripheral “washout”	5 (10%)	2 (5%)	0.461
	Delayed central enhancement	41 (82%)	37 (95%)	0.104
	Targetoid appearance on DWI	31 (62%)	28 (72%)	0.332
	Infiltrative appearance	11 (22%)	21 (54%)	0.002
	Marked diffusion restriction	8 (16%)	3 (8%)	0.240
	Necrosis or severe ischemia	30 (60%)	26 (67%)	0.518
	At least one LR-M feature	44 (88%)	38 (97%)	0.103
LI-RADS major features of HCC	Nonrim APHE	12 (24%)	0 (0%)	0.001
	Nonperipheral “washout”	8 (16%)	2 (5%)	0.109
	Enhancing “capsule”	11 (22%)	3 (8%)	0.066
	At least one major feature of HCC	23 (46%)	3 (8%)	<0.001
Ancillary features (AF) favoring malignancy in general	Restricted diffusion	50 (100%)	39 (100%)	NA
	Mild-moderate T2 hyperintensity	50 (100%)	39 (100%)	NA
	Corona enhancement	18 (36%)	30 (77%)	<0.001
	Iron sparing in solid mass	0 (0%)	0 (0%)	NA
	Fat sparing in solid mass	0 (0%)	0 (0%)	NA
	At least one AF feature favoring malignancy in general	50 (100%)	39 (100%)	NA
LI-RADS ancillary features favoring HCC in particular	Intralesional fat	1 (2%)	0 (0%)	0.103
	Blood products	3 (6%)	1 (3%)	0.628
	Nodule-in-nodule	0 (0%)	0 (0%)	NA
	Mosaic architecture	0 (0%)	0 (0%)	NA
	Nonenhancing “capsule”	0 (0%)	0 (0%)	NA
	At least one AF feature favoring HCC in particular	4 (8%)	1 (3%)	0.272
Others	*Lobe*			0.585
	Right	27 (54%)	17 (44%)	
	Left	19 (38%)	19 (49%)	
	Both	4 (8%)	3 (8%)	
	*Location*			0.002
	Subcapsular	34 (68%)	37 (95%)	
	Not subcapsular	16 (32%)	2 (5%)	
	*Shape*			0.001
	Smooth	21 (42%)	4 (10%)	
	Lobulated/irregular	29 (58%)	35 (90%)	
	Liver surface retraction	11 (22%)	17 (44%)	0.03
	Hepatic lobe atrophy	3 (6%)	7 (18%)	0.098
	Satellite lesions	19 (38%)	10 (26%)	0.462
	Lymph node metastasis	12 (24%)	13 (33%)	0.331

APHE, rim arterial phase hyperenhancement; DCE, delayed central enhancement; HBV, chronic hepatitis B virus infection. Data expressed as counts with percentages in parentheses.

**Table 4:** LI-RADS v2018 Categorization of iCCAs in HBV-positive patients.

	High-HCC-risk group (*n*=50)
LR-3	1 (2%)
LR-4	1 (2%)
LR-5	4 (8%)
LR-M	40 (80%)
LR-TIV in association with LR-M mass	4 (8%)

HBV, chronic hepatitis B virus infection. Data expressed as counts with percentages in parentheses.

## Data Availability

The data supporting the results are included within the article and are available from the corresponding author upon request.
